# Editorial: EMDR and the AIP Model: Healing the Scars of Trauma

**DOI:** 10.3389/fpsyt.2024.1469787

**Published:** 2024-09-25

**Authors:** Ehud Oren, İbrahim Gündoğmuş, Alişan Burak Yaşar

**Affiliations:** ^1^ EMDR Institute of Israel, Tel Aviv, Israel; ^2^ Department of Psychiatry, Ankara Etlik City Hospital, Ankara, Türkiye; ^3^ Department of Psychology, İstanbul Gelşim University, İstanbul, Türkiye

**Keywords:** eye movement desensitization and reprocessing (EMDR), adaptive information processing (AIP), trauma, psychotherapy & EMDR, PTSD - posttraumatic stress disorder

In the dynamic world of modern psychotherapy, the development of innovative and effective treatment methods is paramount for mental health professionals. Trauma treatment, in particular, is a focal point as it directly impacts the quality of life of individuals and plays a critical role in maintaining psychological well-being. In this context, Eye Movement Desensitization and Reprocessing (EMDR) and the Adaptive Information Processing (AIP) model have garnered increasing interest and have been increasingly integrated into clinical practice in recent years (1, 2). EMDR, developed by Francine Shapiro, is designed to alleviate symptoms associated with traumatic memories through structured protocols involving eye movements or other forms of bilateral stimulation (1). The AIP model posits that trauma disrupts the brain’s ability to process information, leading to maladaptive memory storage. EMDR facilitates the reprocessing of these memories, promoting adaptive resolution and integration into existing memory networks (2). Therefore, as a result of the increasing interest in EMDR and AIP, it has become the subject of many studies and articles.

We are pleased to present this edition’s Research Topic review on EMDR and the AIP. This Research Topic features a total of 16 articles, contributed by 72 authors, and supported by the active collaboration of a significant number of reviewers who consistently engaged with and contributed to this Research Topic. These articles include 4 reviews, 6 randomized controlled trials (RCT), 4 original research, 1 case report, and 1 hypothesis and theory paper. Moreover, a wide range of psychotherapeutic methods were explored in these articles, including EMDR, AIP (2), Online EMDR, EMDR 2.0, the Flash Technique, TF-CBT, VSDT, the AIP-based Professional Intervention Program, and Visual EMDR Situation. The breadth, depth, and diversity of the submissions in this Research Topic highlight the robust nature of research on EMDR in both academic settings and clinical practice. These studies serve as an essential resource for professionals seeking new approaches to trauma therapy. In addition, this Research Topic covered not only trauma and PTSD but also on the effectiveness of EMDR and AIP in treating fibromyalgia, intellectual disabilities, personality disorder, prolonged grief, and obsessive symptoms. A large number of innovative articles offer a deeper understanding of EMDR’s contribution to the healing process for post-traumatic and other mental health symptoms, as well as the novel insights provided by the AIP model in understanding the human mind.

A RCT by Zat Çiftçi et al. evaluated the efficacy of EMDR in the treatment of patients with fibromyalgia. The results supported the use of EMDR as an effective treatment for fibromyalgia, particularly in reducing pain and improving sleep and trauma-related symptoms. Thus, this study recommended incorporating EMDR into treatment plans for fibromyalgia patients to enhance physical and psychological outcomes.

Another RCT conducted by Miccoli and Poli supported the efficacy of EMDR as a therapeutic intervention for both post-traumatic and obsessive-compulsive symptoms, especially in the context of COVID-19-related trauma. The study highlighted the importance of addressing bodily sensations and emotions, such as guilt and shame, during EMDR therapy to achieve significant symptom improvement. The study also found that reducing bodily discomfort plays a crucial role in symptom remission, and targeting disgust-related memories could be particularly beneficial in reducing obsessive-compulsive symptoms.

A study by Matthijssen et al. compared the effectiveness of Visual Schema Displacement Therapy (VSDT) with EMDR therapy and a waitlist control group in treating Post-Traumatic Stress Disorder (PTSD). The study suggested both therapies result in improvements in depressive symptoms and general psychopathology, with EMDR demonstrating slightly more favorable outcomes in these areas compared to VSDT. The study supported the use of VSDT as an effective alternative to EMDR for treating PTSD, as both therapies produced similar improvements in PTSD symptoms and overall mental health.

A case report by Matthijssen and Menses from the Altrecht Academic Anxiety Centre demonstrated both the feasibility and effectiveness of an intensive online trauma treatment combining prolonged exposure and EMDR 2.0 for a patient with severe PTSD. The patient achieved full remission of PTSD symptoms, indicating that intensive online trauma treatments can be as effective as in-person sessions.

An article by Rydberg et al. presented a narrative review exploring seven theoretical models, each providing different perspectives on the mechanisms underlying EMDR’s effectiveness and expanding on Shapiro’s original AIP model. While some models focus on psychological processes, others delve into neural mechanisms, or a combination of both. Authors generally agree that Bilateral/Dual Attention Stimulation (BL/DAS), such as eye movements, is not solely responsible for EMDR’s therapeutic effects; instead, a combination of EMDR principles, procedures, and protocols plays a crucial role.

Aiming to provide neuroanatomical evidence for the effects of EMDR using a rat model of Acute Variable Stress (AVS), the study conducted by Ruvalcaba-Delgadillo et al. found that visual EMDR stimulation attenuated stress-induced dendritic atrophy in hippocampal neurons. This study offers compelling evidence that EMDR may have protective effects on brain structure, underscoring its potential in treating trauma-related disorders by restoring neuronal integrity under stress.

An article by Spicer is a mini-review of the theory, research, and application of EMDR therapy for prolonged grief. Accordingly, EMDR also holds promise in the treatment of prolonged grief. Integrating the dual process model of coping with bereavement, EMDR helps individuals reprocess distressing grief-related memories, facilitating adaptive coping and emotional resolution. This approach can be particularly beneficial for individuals struggling with complicated grief symptoms that interfere with daily functioning.

A RCT by Alting van Geusau et al. compared the comparative effectiveness, efficiency, and acceptability of various approaches in the treatment of PTSD. In an effort to enhance current treatment options, the research investigated EMDR therapy, a novel adaptation known as EMDR 2.0, and an innovative intervention called the Flash technique. In a sample of 130 individuals diagnosed with PTSD, this study aimed to identify differences in symptom alleviation and explore the moderators of treatment effectiveness. The results of the study advance current treatment options for PTSD and offer therapists alternative treatment methods based on factors such as effectiveness, efficiency, and acceptability.

A review by Kaptan et al. focused on the evidence surrounding the remote delivery of EMDR therapy, which emerges as a crucial response to the growing demand for mental health support. By systematically reviewing studies that assess the efficacy of online EMDR therapy, this paper evaluated its potential as a viable treatment option for reducing symptoms of PTSD, anxiety, and depression during a period marked by limited in-person interaction.

One of the articles by De Jongh featured in this Research Topic focused on the cumulative impact of traumatic events on individuals and how these experiences can contribute to the development of personality disorders. The article highlights that adverse childhood experiences (ACEs) can lead to the emergence of personality disorders, which are characterized by significant difficulties in emotional regulation, self-concept, cognitive functions, and interpersonal relationships. Given the often lengthy and costly nature of traditional treatments for these disorders, exploring alternative and complementary approaches is increasingly necessary. This paper presented a treatment approach grounded in Shapiro’s AIP model, utilizing EMDR therapy to process traumatic memories. The article examined the current empirical support for this approach and offered practical guidance on developing a case conceptualization for treating personality disorders with EMDR therapy, illustrated by a case example.

A review by Schipper-Eindhoven et al. sought to systematically identify and categorize the difficulties encountered in applying EMDR to people with intellectual disabilities and the adaptations that therapists made to overcome these difficulties. EMDR therapy is successfully adapted for individuals with intellectual disabilities, demonstrating its versatility and effectiveness across diverse populations. This article highlighted the positive outcomes of these adaptations, demonstrating that EMDR can be effectively tailored to meet the unique needs of this group, facilitating significant psychological improvements.

A RCT by Molero-Zafra et al. compared the effects of Trauma-Focused Cognitive Behavioral Therapy (TF-CBT) and EMDR in women who experienced childhood sexual abuse. The research found that both therapies significantly reduce trauma symptoms and improve quality of life. TF-CBT led to improvements in emotional regulation, reexperiencing, and avoidance, while the EMDR group showed significant progress in dissociation and perceived quality of life. The study supported the feasibility of both approaches in an online group format and their effectiveness in treating trauma.

A RCT by Burback et al. provided preliminary evidence that web-based EMDR therapy is a viable and safe approach for reducing suicidal ideation and associated symptoms in a complex psychiatric population. Participants in the EMDR group showed significant reductions in suicidal ideation, depression, anxiety, and post-traumatic stress symptoms. The authors suggest that EMDR can be an effective transdiagnostic treatment for suicidal ideation, offering a promising alternative to traditional approaches.

A study by Kaptan et al. evaluated the implementation of online EMDR within a primary care network in England, finding significant improvements in patients’ anxiety, depression, and PTSD symptoms. Many patients described the therapy as life-changing, highlighting its effectiveness in reducing distressing symptoms and improving overall mental health. Patients reported that the therapy provided them with coping strategies, improved their sleep, and reduced the frequency of nightmares and flashbacks.

The study by Manfield et al. on the efficacy of the Flash Technique (FT) evaluated its acceptability, safety, and effectiveness as a method designed to reduce the intensity of disturbing memories with minimal distress during the process. The research included four similar studies conducted across different settings with a total of 654 participants. These results suggested a significant reduction in the disturbance levels associated with traumatic memories across all settings. On average, participants experienced a reduction in distress of more than two-thirds after just 15 minutes of FT. Therefore, results showed that FT could be a highly effective and efficient method for addressing traumatic memories in both clinical and non-clinical settings.

A study by Woldemariam et al. evaluated the effectiveness of the Professional Intervention Program for Adversity (PIPA), a group-based intervention designed to reduce trauma and stress using principles from EMDR therapy and the AIP model. The program was administered to over 220 individuals traumatized by the civil war in Ethiopia. Participants reported feeling more connected, positive, and engaged following the program, indicating its effectiveness in fostering group cohesion and reducing trauma-related distress.

These important and scientifically valuable studies demonstrate the powerful application and significant developmental potential of EMDR therapy. We hope that the valuable findings from both case studies and research will be replicated by different researchers, thus solidifying our knowledge. The abundance and quality of the articles in this Research Topic support the growing use of EMDR Therapy in the fields of both mental health and general health, and it being recommended in multiple treatment guidelines.

## References

[1] Shapiro F. Eye Movement Desensitization and Reprocessing (EMDR) Therapy: Basic Principles, Protocols, and Procedures. New York: Guilford Publications (2017).

[2] Hase M Balmaceda UM Ostacoli L Liebermann P Hofmann A. The AIP model of EMDR therapy and pathogenic memories. Frontiers Psychology (2017) 8:1578. doi: 10.3389/fpsyg.2017.01578 PMC5613256 28983265

